# 3,6,9,16,19,22-Hexaaza­tricyclo­[22.2.2.2^11,14^]­triaconta-1(27),11 (30),12,14(29),24(28),25-hexa­ene hexa­kis(*p*-toluene­sulfonate) dihydrate

**DOI:** 10.1107/S1600536809035648

**Published:** 2009-09-09

**Authors:** Musabbir A. Saeed, Jameskia J. Thompson, Frank R. Fronczek, Md. Alamgir Hossain

**Affiliations:** a Department of Chemistry and Biochemistry, Jackson State University, MS 39217, USA; b Department of Chemistry, Louisiana State University, Baton Rouge, LA 70803, USA

## Abstract

In the title compound, C_24_H_44_N_6_^6+^·6C_7_H_7_O_3_S^−^·2H_2_O, the macrocycle crystallizes in its hexa­protonated form, accompanied by six *p*-toluene­sulfonate ions and two water mol­ecules, and lies on an inversion center. The three independent *p*-toluene­sulfonate anions and their inversion equivalents at (1 − *x*, 1 − *y*, 1 − *z*) are linked to the macrocyclic cation through N—H⋯O hydrogen bonds. Of these, two *p*-toluene­sulfonate ions are located on opposite sides of the macrocyclic plane and are linked to bridgehead N atoms *via* N—H⋯O hydrogen bonds. The remaining four *p*-toluene­sulfonate ions bridge two adjacent macrocyclic cationic units through N—H⋯O hydrogen bonding involving other N atoms, forming a chain along the *a* axis. The water mol­ecules, which could not be located and may be disordered, do not inter­act with the macrocycle; however, they form hydrogen bonds with anions.

## Related literature

For general background, see: Bianchi *et al.* (1997 ▶); Chen & Martell (1991 ▶); Hossain (2008 ▶); Llobet *et al.* (1994 ▶); Nagarajan & Ganem (1987 ▶); Ragunathan & Schneider (1996 ▶). For related structures, see: Bazzicalupi *et al.* (1995 ▶); Clifford *et al.* (2001 ▶); He *et al.* (2000 ▶); Li *et al.* (2009 ▶); Liu *et al.* (2008 ▶); Lu *et al.* (1995 ▶, 1998 ▶); Zhu *et al.* (2002 ▶).
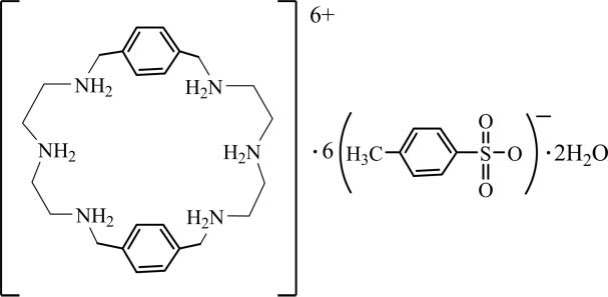


## Experimental

### 

#### Crystal data

C_24_H_44_N_6_^6+^·6C_7_H_7_O_3_S^−^·2H_2_O
*M* *_r_* = 1479.80
Triclinic, 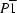

*a* = 11.513 (3) Å
*b* = 12.639 (5) Å
*c* = 13.556 (6) Å
α = 78.578 (16)°
β = 71.88 (2)°
γ = 89.30 (2)°
*V* = 1835.0 (12) Å^3^
*Z* = 1
Mo *K*α radiation
μ = 0.26 mm^−1^
*T* = 90 K
0.17 × 0.10 × 0.03 mm

#### Data collection

Nonius KappaCCD diffractometer with an Oxford Cryosystems Cryostream cooler
Absorption correction: multi-scan (*SCALEPACK*; Otwinowski & Minor, 1997 ▶) *T*_min_ = 0.947, *T*_max_ = 0.992
27691 measured reflections
7213 independent reflections
2920 reflections with *I* > 2σ(*I*)
*R*_int_ = 0.145

#### Refinement

*R*[*F* ^2^ > 2σ(*F* ^2^)] = 0.072
*wR*(*F* ^2^) = 0.156
*S* = 0.97
7213 reflections
446 parameters
H-atom parameters constrained
Δρ_max_ = 0.39 e Å^−3^
Δρ_min_ = −0.33 e Å^−3^

### 

Data collection: *COLLECT* (Nonius, 1999 ▶); cell refinement: *DENZO* and *SCALEPACK* (Otwinowski & Minor, 1997 ▶); data reduction: *DENZO* and *SCALEPACK*; program(s) used to solve structure: *SIR92* (Altomare *et al.*, 1994 ▶); program(s) used to refine structure: *SHELXL97* (Sheldrick, 2008 ▶); molecular graphics: *ORTEP-3 for Windows* (Farrugia, 1997 ▶); software used to prepare material for publication: *SHELXL97*.

## Supplementary Material

Crystal structure: contains datablocks global, I. DOI: 10.1107/S1600536809035648/ci2898sup1.cif

Structure factors: contains datablocks I. DOI: 10.1107/S1600536809035648/ci2898Isup2.hkl

Additional supplementary materials:  crystallographic information; 3D view; checkCIF report

## Figures and Tables

**Table 1:** Hydrogen-bond geometry (Å, °)

*D*—H⋯*A*	*D*—H	H⋯*A*	*D*⋯*A*	*D*—H⋯*A*
N1—H11N⋯O7^i^	0.92	1.86	2.738 (5)	159
N1—H12N⋯O8	0.92	2.10	2.923 (6)	148
N1—H12N⋯O9	0.92	2.33	3.130 (5)	145
N2—H21N⋯O5	0.92	1.85	2.745 (5)	164
N2—H22N⋯O1^ii^	0.92	1.83	2.706 (4)	159
N3—H31N⋯O4^ii^	0.92	1.84	2.748 (4)	168
N3—H32N⋯O2	0.92	1.92	2.842 (5)	177

Symmetry codes: (i) 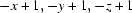; (ii) 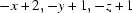.

**Fig. 1.**
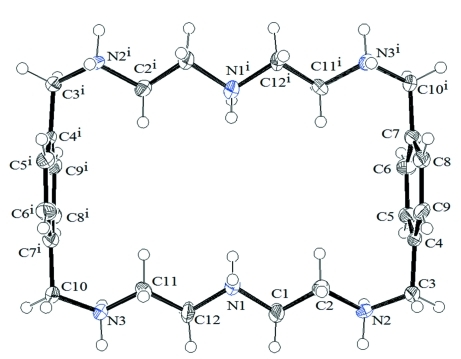
The molecular structure of cationic unit in (1). Displacement ellipsoids are drawn at the 50% probability level. [Symmetry codes: (i) 1 - x, 1 - y, 1 - z].

**Fig. 2.**
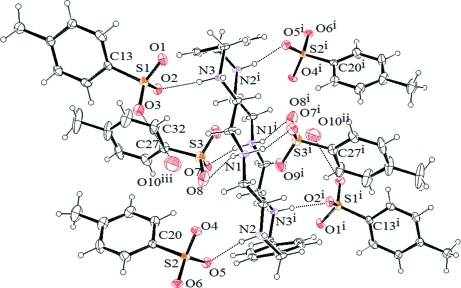
The molecular structure of (1) showing the atom-numbering scheme and hydrogen bonding interactions. Displacement ellipsoids are drawn at the 50% probability level. [Symmetry codes: (i) 1 - x, 1 - y, 1 - z; (ii) 2 - x, 1 - y, 1 - z.]

**Fig. 3.**
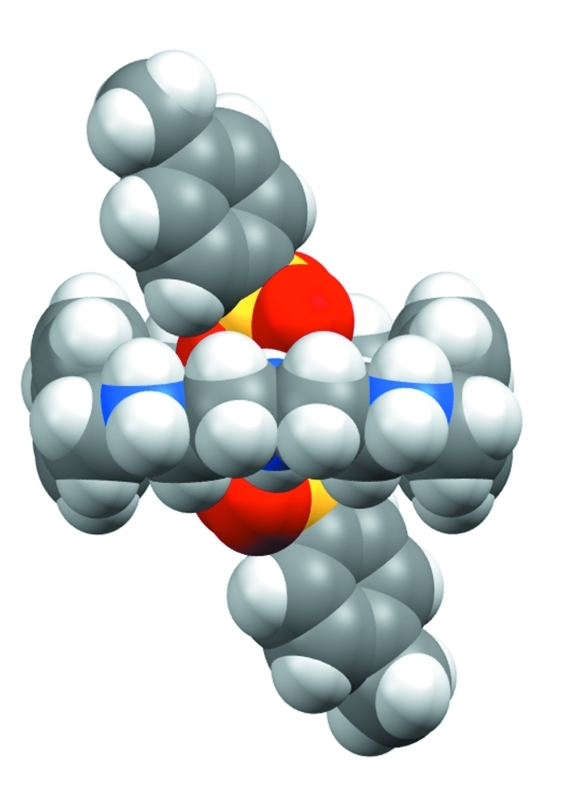
Space-filling view of (1) showing two anions bonded with macrocycle at the both faces.

**Fig. 4.**
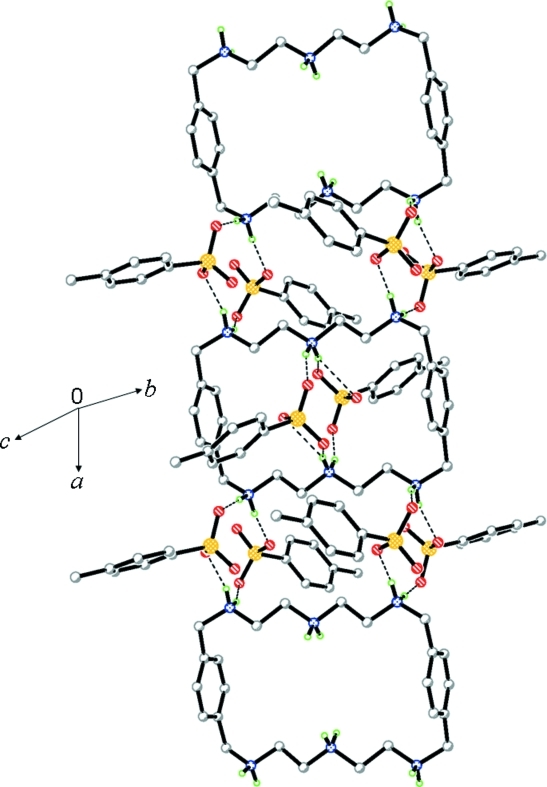
View of a hydrogen-bonded (dashed lines) chain in (1).

## supplementary crystallographic information

### Comment

Azamacrocyles mimic many natural polyamines including putrescine, spermidine and spermine, that are known to complex negatively charged nucleotides through hydrogen bonding interactions with phosphate groups (Nagarajan & Ganem, 1987; Bianchi *et al.*, 1997)). Simple hexaazamacrocycles which are conveniently synthesized from Schiff base derived reaction (Chen & Martell, 1991) are known to complex both anions and cations (Llobet *et al.*, 1994; Hossain, 2008). For example, *m*-xylyl macrocycle with six N atoms and rigid spacers were shown to form complexes with sulfate (Clifford *et al.*, 2001), pyrophosphate (Lu *et al.*, 1995), and triphosphate (Lu *et al.*, 1998). A *m*-xylyl analogue with propylene chains, was recently reported to complex with chloride showing a chair-like structure, where chloride anions are bonded to protonated amines outside the cavity (Liu *et al.*, 2008). The macrocycle in the title compound containing slightly larger cavity with *p*-xylyl groups, was reported to bind perchlorate (Bazzicalupi, *et al.*, 1995) and benzene-1,2,4,5-tetracarboxylate ((Zhu *et al.*, 2002) in the tetraprotonated form. Incorporating metal ions, this compound was found to catalyze the hydrolysis of bis(*p*-nitrophenyl)-phosphate (Ragunathan & Schneider, 1996), and was also shown to bind calf thymus DNA (Li *et al.*, 2009). As a part of our work in the field of anion recognition, we synthesized the *p*-xylyl based macrocycle and isolated crystals of the *p*-toluenesulfonate salt. This report describes the synthesis and structural aspect of a ditopic anion complex of *p*-xylyl macrocycle.

Single crystal analysis of the *p*-toluenesulfonate salt reveals that the ligand is hexaprotonated and crystallized with six *p*-toluenesulfonate anions and two water molecules. Each protonated amine of the macrocycle is involved in coordinating one *p*-toluenesulfonate with short (N···O) hydrogen bonds (Table 1). The cationic macrocycle lies on an inversion center. The macrocycle is found to adopt a rectangular shape in which two aromatic units are parallel to each other with a centroid-to-centroid distance of 9.990 Å (Fig. 1). The shape of the macrocycle is quite different from those observed in the neutral macrocycle (He *et al.*, 2000) or its larger cationic analogue with *m*-xylyl spacers in the chloride complex (Liu *et al.*, 2008), both of which adopt a chair conformation with two flip-flop aromatic moieties. In the solid state, the hydrogen atoms on the bridgehead amines direct toward the cavity, while those on the remaining nitrogen atoms point outwards from the cavity to minimize the electrostatic repulsion forces. The NCCN chains (N1—C1—C2—N2, N3—C11—C12—N1 and their symmetry related counterparts) linking the bridgehead N atoms, are essentially unstrained, with the torsion angles of -172.0 (4) and 170.9 (4)°, close to the *trans* conformation (*ca* 180°). On the other hand, *gauche* conformations (*ca* 60°) were found for the CNCC chains (C2—N2—C3—C4, C11—N3—C10—C7^i^ and their counterparts) associated with the aromatic rings, with torsion angles of -51.8 (5) and 51.9 (5)°.

All six *p*-toluenesulfonate anions are strongly bonded to both faces of the macrocycle, with three groups at each face (Fig. 2). Four *p*-toluenesulfonates are singly bonded to the protonated amines (N2, N2^i^, N3 and N3^i^) linking the aromatic spacers, with almost linear N—H···O interactions (164 to 177 °). The distances in N···O bonds are in the range from 2.706 (4) to 2.842 (5) Å. Two other anionic groups are located above and below the macrocyclic plane, each with two N···O bonds with the central amines (N1 and N1^i^). In this case two oxygen atoms, O7 and O8 and their symmetry related O7^ii^ and O8^ii^ act as hydrogen bond acceptors and bridge the amines from the opposite sides. These two anions are partially included in the cavity with a ditopic binding mode (Fig. 3). In the crystal structure, the ionic units are linked by N—H..O hydrogen bonds forming a chain along the *a* axis. Along the *c* axis, the C4-C9 aromatic rings of the adjacent chains are stacked, with a centroid-to-centroid distance of 4.533 (3) Å (Fig. 4). The anions are found within the channels formed by the macrocycles, facing in opposite directions alternatively and are linked to the macrocycles with extensive hydrogen bonding frameworks.

In summary, a novel ditopic complex of *p*-toluenesulfonate anion has been synthesized, and its structure has been presented. The surrounding *p*-toluenesulfonates act as hydrogen bond acceptors for the nitrogen sites in the macrocycle, and play an important role in forming a hydrogen bonding network in the three dimensional structure. The water molecules do not interact with the macrocycle, however they form hydrogen bonds with anions.

### Experimental

The synthesis was carried out following a modified literature procedure (Chen & Martell, 1991). An equimolar amount of diethylenetriamine (1.00 g, 9.70 *x* 10 ^-3^ mol) in CH_3_OH (200 ml) and terephthalaldehyde (1.30 g, 9.70 *x* 10 ^-3^ mol) in CH_3_OH (200 ml) were added to CH_3_OH (400 ml) over 4 h at 0°C. The mixture was stirred at room temperature for another 24 h. After the solvent was evaporated, the Schiff base was reduced to amine with NaBH_4_ (1.73 g, 45.7 *x* 10 ^-3^ mol) in CH_3_OH (100 ml) at room temperature for 12 h. After evaporating the solvent under reduced pressure, the residue was dissolved in 1 *M* aq NaOH solution (100 ml), and the aqueous phase was extracted by CH_2_Cl_2_ (3x100 ml). The organic layers were combined and dried with MgSO_4_. Evaporation of the solvent gave a white powder of the neutral macrocycle. Yield: 60%. ^1^H NMR (300 MHz, CDCl_3_, TMS): δ 7.29 (s, 8H, ArH), 3.80 (s, 8H, ArCH_2_), 2.85 (t, 8H, CH_2_), 2.83 (t, 8H, CH_2_). The *p*-toluenesulfonate salt was obtained by titrating the macrocycle (50 mg) dissolved in CH_3_OH (2 ml) with TsOH. The addition of diethyl ether (2 ml) yielded a white precipitate of *p*-toluenesulfonate salt that was filtered and dried. Yield: 80%. ^1^H NMR (300 MHz, D_2_O, TSP): δ 7.70 (d, 12H, ArH), 7.42 (d, 12H, ArH), 7.26 (s, 8H, ArH), 4.21 (s, 8H, ArCH_2_), 3.27 (t, 8H, CH_2_), 3.01 (t, 8H, CH_2_), 2.43 (s, 12H, CH_2_). Single crystals suitable for X-ray analysis were obtained by slow evaporation of the solvent.

### Refinement

H atoms on C were placed in idealized positions with C-H distances of 0.95-0.99 Å and thereafter treated as riding. Those on N were all visible in difference maps, but were placed in idealized positions with a N-H distance of 0.92 Å. *U*_iso_(H) values were assigned as 1.2 times *U*_eq_ of the attached atom (1.5 for methyl). A torsional parameter was refined for each methyl group. H atoms on the water molecule could not be located.

### Crystal data

**Table:**

C_24_H_44_N_6_^6+^·6C_7_H_7_O_3_S^−^·2H_2_O	*Z* = 1
*M**_r_* = 1479.80	*F*(000) = 784
Triclinic, *P*1	*D*_x_ = 1.339 Mg m^−^^3^
Hall symbol: -P 1	Mo *K*α radiation, λ = 0.71073 Å
*a* = 11.513 (3) Å	Cell parameters from 6760 reflections
*b* = 12.639 (5) Å	θ = 2.5–26.0°
*c* = 13.556 (6) Å	µ = 0.26 mm^−^^1^
α = 78.578 (16)°	*T* = 90 K
β = 71.88 (2)°	Plate, colorless
γ = 89.30 (2)°	0.17 × 0.10 × 0.03 mm
*V* = 1835.0 (12) Å^3^	

### Data collection

**Table:**

Nonius KappaCCD diffractometer with an Oxford Cryosystems Cryostream cooler	7213 independent reflections
Radiation source: fine-focus sealed tube	2920 reflections with *I* > 2σ(*I*)
graphite	*R*_int_ = 0.145
ω and φ scans	θ_max_ = 26.3°, θ_min_ = 2.7°
Absorption correction: multi-scan (*SCALEPACK*; Otwinowski & Minor, 1997)	*h* = −13→14
*T*_min_ = 0.947, *T*_max_ = 0.992	*k* = −15→15
27691 measured reflections	*l* = −16→16

### Refinement

**Table:**

Refinement on *F*^2^	Secondary atom site location: difference Fourier map
Least-squares matrix: full	Hydrogen site location: inferred from neighbouring sites
*R*[*F*^2^ > 2σ(*F*^2^)] = 0.072	H-atom parameters constrained
*wR*(*F*^2^) = 0.156	*w* = 1/[σ^2^(*F*_o_^2^) + (0.0515*P*)^2^] where *P* = (*F*_o_^2^ + 2*F*_c_^2^)/3
*S* = 0.97	(Δ/σ)_max_ = 0.001
7213 reflections	Δρ_max_ = 0.39 e Å^−^^3^
446 parameters	Δρ_min_ = −0.33 e Å^−^^3^
0 restraints	Extinction correction: *SHELXL97* (Sheldrick, 2008), Fc^*^=kFc[1+0.001xFc^2^λ^3^/sin(2θ)]^-1/4^
Primary atom site location: structure-invariant direct methods	Extinction coefficient: 0.0019 (7)

### Special details

**Table:**

Refinement. Refinement of *F*^2^ against ALL reflections. The weighted *R*-factor *wR* and goodness of fit *S* are based on *F*^2^, conventional *R*-factors *R* are based on *F*, with *F* set to zero for negative *F*^2^. The threshold expression of *F*^2^ > σ(*F*^2^) is used only for calculating *R*-factors(gt) *etc*. and is not relevant to the choice of reflections for refinement. *R*-factors based on *F*^2^ are statistically about twice as large as those based on *F*, and *R*- factors based on ALL data will be even larger.

### Fractional atomic coordinates and isotropic or equivalent isotropic displacement parameters (Å^2^)

**Table:**

	*x*	*y*	*z*	*U*_iso_*/*U*_eq_	
N1	0.7392 (3)	0.5311 (3)	0.4777 (3)	0.0292 (10)	
H11N	0.7039	0.5968	0.4705	0.035*	
H12N	0.6798	0.4779	0.4892	0.035*	
N2	0.8843 (3)	0.5451 (3)	0.1864 (3)	0.0242 (10)	
H21N	0.8920	0.4720	0.1907	0.029*	
H22N	0.9589	0.5751	0.1815	0.029*	
N3	0.7276 (3)	0.5161 (3)	0.7591 (3)	0.0222 (9)	
H31N	0.8044	0.5469	0.7455	0.027*	
H32N	0.7331	0.4423	0.7725	0.027*	
C1	0.8391 (4)	0.5271 (4)	0.3779 (3)	0.0331 (13)	
H1A	0.8667	0.4525	0.3792	0.040*	
H1B	0.9097	0.5751	0.3710	0.040*	
C2	0.7921 (4)	0.5632 (4)	0.2853 (3)	0.0263 (12)	
H2A	0.7146	0.5220	0.2974	0.032*	
H2B	0.7753	0.6409	0.2782	0.032*	
C3	0.8538 (4)	0.5918 (4)	0.0876 (4)	0.0281 (12)	
H3A	0.8608	0.6717	0.0750	0.034*	
H3B	0.9132	0.5688	0.0264	0.034*	
C4	0.7271 (4)	0.5565 (4)	0.0956 (3)	0.0219 (11)	
C5	0.6934 (4)	0.4486 (4)	0.1162 (3)	0.0276 (12)	
H5	0.7526	0.3962	0.1200	0.033*	
C6	0.5747 (4)	0.4155 (4)	0.1315 (4)	0.0286 (12)	
H6	0.5525	0.3404	0.1469	0.034*	
C7	0.4869 (4)	0.4901 (4)	0.1246 (3)	0.0245 (12)	
C8	0.5205 (4)	0.5989 (4)	0.1009 (3)	0.0253 (12)	
H8	0.4624	0.6513	0.0933	0.030*	
C9	0.6402 (4)	0.6312 (4)	0.0885 (3)	0.0247 (12)	
H9	0.6625	0.7060	0.0748	0.030*	
C10	0.6439 (3)	0.5474 (4)	0.8560 (3)	0.0239 (12)	
H10A	0.6493	0.6270	0.8477	0.029*	
H10B	0.6698	0.5149	0.9180	0.029*	
C11	0.6896 (4)	0.5483 (4)	0.6635 (3)	0.0246 (12)	
H11A	0.6815	0.6275	0.6486	0.030*	
H11B	0.6092	0.5124	0.6751	0.030*	
C12	0.7840 (4)	0.5161 (5)	0.5708 (4)	0.0389 (15)	
H12A	0.8608	0.5607	0.5523	0.047*	
H12B	0.8019	0.4394	0.5905	0.047*	
S1	0.86065 (10)	0.27995 (10)	0.83408 (10)	0.0307 (4)	
O1	0.8820 (2)	0.3732 (2)	0.8763 (3)	0.0301 (9)	
O2	0.7479 (2)	0.2887 (2)	0.8063 (2)	0.0321 (9)	
O3	0.9661 (3)	0.2628 (3)	0.7468 (3)	0.0405 (10)	
C13	0.8414 (4)	0.1649 (4)	0.9368 (4)	0.0239 (12)	
C14	0.8237 (4)	0.0639 (4)	0.9169 (4)	0.0368 (14)	
H14	0.8200	0.0575	0.8493	0.044*	
C15	0.8115 (4)	−0.0272 (4)	0.9950 (4)	0.0385 (14)	
H15	0.7995	−0.0961	0.9805	0.046*	
C16	0.8164 (4)	−0.0201 (4)	1.0952 (4)	0.0298 (13)	
C17	0.8351 (4)	0.0810 (4)	1.1128 (4)	0.0349 (13)	
H17	0.8388	0.0876	1.1803	0.042*	
C18	0.8485 (4)	0.1730 (4)	1.0349 (4)	0.0370 (14)	
H18	0.8625	0.2417	1.0488	0.044*	
C19	0.8042 (4)	−0.1200 (4)	1.1794 (4)	0.0389 (14)	
H19A	0.8052	−0.0996	1.2452	0.058*	
H19B	0.8725	−0.1659	1.1561	0.058*	
H19C	0.7267	−0.1599	1.1919	0.058*	
S2	1.03881 (10)	0.29487 (10)	0.21857 (10)	0.0261 (4)	
O4	1.0570 (2)	0.3649 (2)	0.2876 (2)	0.0265 (8)	
O5	0.9472 (2)	0.3379 (2)	0.1689 (2)	0.0310 (9)	
O6	1.1516 (2)	0.2716 (2)	0.1447 (2)	0.0318 (9)	
C20	0.9732 (4)	0.1722 (4)	0.3034 (4)	0.0243 (12)	
C21	1.0362 (4)	0.0795 (4)	0.3002 (4)	0.0413 (14)	
H21	1.1164	0.0814	0.2517	0.050*	
C22	0.9831 (5)	−0.0177 (4)	0.3677 (5)	0.0515 (16)	
H22	1.0270	−0.0817	0.3641	0.062*	
C23	0.8652 (5)	−0.0217 (5)	0.4410 (4)	0.0444 (15)	
C24	0.8042 (4)	0.0714 (5)	0.4437 (4)	0.0429 (15)	
H24	0.7245	0.0699	0.4930	0.052*	
C25	0.8559 (4)	0.1684 (4)	0.3760 (4)	0.0344 (13)	
H25	0.8113	0.2321	0.3791	0.041*	
C26	0.8091 (5)	−0.1286 (4)	0.5137 (5)	0.0640 (19)	
H26A	0.7470	−0.1148	0.5774	0.096*	
H26B	0.8731	−0.1706	0.5340	0.096*	
H26C	0.7709	−0.1694	0.4765	0.096*	
S3	0.51388 (11)	0.31199 (11)	0.53979 (12)	0.0374 (4)	
O7	0.4106 (3)	0.2991 (3)	0.5021 (3)	0.0422 (10)	
O8	0.6303 (3)	0.3321 (3)	0.4573 (3)	0.0696 (14)	
O9	0.4946 (3)	0.3943 (3)	0.6041 (3)	0.0540 (11)	
C27	0.5184 (4)	0.1876 (4)	0.6248 (4)	0.0338 (13)	
C28	0.4736 (4)	0.0918 (4)	0.6118 (5)	0.0427 (15)	
H28	0.4402	0.0912	0.5560	0.051*	
C29	0.4784 (5)	−0.0029 (5)	0.6816 (6)	0.061 (2)	
H29	0.4467	−0.0685	0.6733	0.073*	
C30	0.5274 (5)	−0.0056 (5)	0.7628 (6)	0.070 (2)	
C31	0.5708 (5)	0.0922 (5)	0.7738 (5)	0.075 (2)	
H31	0.6030	0.0934	0.8302	0.089*	
C32	0.5681 (4)	0.1871 (5)	0.7050 (5)	0.0520 (17)	
H32	0.6006	0.2526	0.7127	0.062*	
C33	0.5288 (6)	−0.1102 (5)	0.8403 (7)	0.118 (4)	
H33A	0.5662	−0.1657	0.8009	0.177*	
H33B	0.4448	−0.1346	0.8840	0.177*	
H33C	0.5764	−0.0978	0.8860	0.177*	
O10	1.0391 (4)	0.6764 (4)	0.4660 (4)	0.0990 (16)	

### Atomic displacement parameters (Å^2^)

**Table:**

	*U*^11^	*U*^22^	*U*^33^	*U*^12^	*U*^13^	*U*^23^
N1	0.024 (2)	0.042 (3)	0.019 (2)	0.0028 (19)	−0.0035 (19)	−0.009 (2)
N2	0.0124 (19)	0.037 (3)	0.022 (2)	0.0006 (17)	−0.0023 (17)	−0.007 (2)
N3	0.0195 (19)	0.028 (2)	0.018 (2)	−0.0020 (17)	−0.0027 (17)	−0.0059 (19)
C1	0.023 (3)	0.056 (4)	0.021 (3)	0.007 (2)	−0.005 (2)	−0.013 (3)
C2	0.021 (2)	0.033 (3)	0.024 (3)	0.000 (2)	−0.003 (2)	−0.011 (3)
C3	0.018 (2)	0.048 (3)	0.018 (3)	−0.001 (2)	−0.005 (2)	−0.006 (3)
C4	0.014 (2)	0.036 (3)	0.015 (3)	0.001 (2)	−0.001 (2)	−0.011 (2)
C5	0.019 (3)	0.039 (4)	0.027 (3)	0.006 (2)	−0.006 (2)	−0.013 (3)
C6	0.028 (3)	0.027 (3)	0.031 (3)	−0.007 (2)	−0.009 (2)	−0.005 (3)
C7	0.020 (3)	0.035 (3)	0.022 (3)	0.004 (2)	−0.008 (2)	−0.010 (3)
C8	0.021 (3)	0.034 (3)	0.021 (3)	0.005 (2)	−0.006 (2)	−0.007 (2)
C9	0.022 (3)	0.030 (3)	0.019 (3)	−0.007 (2)	0.000 (2)	−0.006 (2)
C10	0.021 (3)	0.034 (3)	0.016 (3)	0.000 (2)	−0.004 (2)	−0.006 (2)
C11	0.023 (2)	0.033 (3)	0.022 (3)	0.002 (2)	−0.013 (2)	−0.005 (2)
C12	0.025 (3)	0.075 (4)	0.015 (3)	0.009 (3)	−0.005 (2)	−0.010 (3)
S1	0.0238 (7)	0.0303 (9)	0.0379 (9)	0.0036 (6)	−0.0102 (6)	−0.0060 (7)
O1	0.0245 (17)	0.022 (2)	0.047 (2)	−0.0024 (14)	−0.0159 (16)	−0.0081 (17)
O2	0.0287 (18)	0.029 (2)	0.042 (2)	0.0011 (15)	−0.0177 (16)	−0.0050 (17)
O3	0.0298 (19)	0.044 (2)	0.038 (2)	0.0123 (16)	−0.0013 (17)	−0.0027 (18)
C13	0.016 (2)	0.024 (3)	0.033 (3)	0.004 (2)	−0.006 (2)	−0.009 (3)
C14	0.052 (3)	0.033 (4)	0.035 (4)	−0.003 (3)	−0.020 (3)	−0.017 (3)
C15	0.047 (3)	0.026 (4)	0.044 (4)	0.000 (3)	−0.014 (3)	−0.013 (3)
C16	0.025 (3)	0.028 (4)	0.036 (4)	0.003 (2)	−0.010 (2)	−0.007 (3)
C17	0.045 (3)	0.029 (4)	0.030 (3)	0.002 (3)	−0.014 (3)	−0.003 (3)
C18	0.036 (3)	0.032 (4)	0.052 (4)	−0.003 (2)	−0.018 (3)	−0.022 (3)
C19	0.039 (3)	0.039 (4)	0.039 (4)	0.002 (3)	−0.012 (3)	−0.006 (3)
S2	0.0209 (7)	0.0306 (8)	0.0250 (8)	−0.0015 (6)	−0.0034 (6)	−0.0079 (7)
O4	0.0207 (16)	0.031 (2)	0.030 (2)	−0.0027 (14)	−0.0065 (14)	−0.0141 (17)
O5	0.0295 (18)	0.035 (2)	0.034 (2)	0.0029 (15)	−0.0168 (16)	−0.0073 (17)
O6	0.0240 (17)	0.039 (2)	0.024 (2)	0.0006 (15)	0.0047 (15)	−0.0082 (17)
C20	0.024 (3)	0.027 (3)	0.026 (3)	0.001 (2)	−0.011 (2)	−0.010 (2)
C21	0.031 (3)	0.035 (4)	0.049 (4)	−0.001 (3)	0.001 (3)	−0.011 (3)
C22	0.056 (4)	0.025 (4)	0.068 (5)	0.004 (3)	−0.017 (3)	−0.001 (3)
C23	0.039 (3)	0.041 (4)	0.052 (4)	−0.016 (3)	−0.021 (3)	0.006 (3)
C24	0.031 (3)	0.047 (4)	0.043 (4)	−0.011 (3)	−0.008 (3)	0.002 (3)
C25	0.024 (3)	0.035 (4)	0.041 (4)	0.002 (2)	−0.009 (3)	−0.004 (3)
C26	0.061 (4)	0.046 (4)	0.080 (5)	−0.022 (3)	−0.030 (3)	0.014 (4)
S3	0.0244 (7)	0.0398 (10)	0.0454 (10)	0.0028 (6)	−0.0109 (7)	−0.0034 (8)
O7	0.040 (2)	0.046 (2)	0.051 (3)	0.0073 (17)	−0.0270 (18)	−0.0139 (19)
O8	0.032 (2)	0.066 (3)	0.075 (3)	0.0132 (19)	0.012 (2)	0.021 (2)
O9	0.075 (3)	0.033 (2)	0.072 (3)	0.0047 (19)	−0.045 (2)	−0.017 (2)
C27	0.022 (3)	0.035 (4)	0.046 (4)	−0.002 (2)	−0.014 (3)	−0.008 (3)
C28	0.031 (3)	0.033 (4)	0.063 (4)	−0.009 (3)	−0.010 (3)	−0.016 (3)
C29	0.039 (3)	0.021 (4)	0.114 (6)	−0.004 (3)	−0.014 (4)	−0.008 (4)
C30	0.051 (4)	0.036 (4)	0.120 (6)	−0.011 (3)	−0.047 (4)	0.023 (4)
C31	0.071 (5)	0.057 (5)	0.102 (6)	−0.025 (4)	−0.059 (4)	0.022 (4)
C32	0.052 (4)	0.034 (4)	0.070 (5)	−0.018 (3)	−0.032 (3)	0.012 (3)
C33	0.106 (6)	0.049 (5)	0.193 (9)	−0.030 (4)	−0.087 (6)	0.058 (6)
O10	0.116 (4)	0.111 (4)	0.087 (4)	0.044 (3)	−0.047 (3)	−0.039 (3)

### Geometric parameters (Å, °)

**Table:**

N1—C12	1.484 (5)	C15—C16	1.397 (7)
N1—C1	1.488 (5)	C15—H15	0.95
N1—H11N	0.92	C16—C17	1.376 (6)
N1—H12N	0.92	C16—C19	1.501 (6)
N2—C2	1.486 (5)	C17—C18	1.381 (6)
N2—C3	1.496 (5)	C17—H17	0.95
N2—H21N	0.92	C18—H18	0.95
N2—H22N	0.92	C19—H19A	0.98
N3—C11	1.476 (5)	C19—H19B	0.98
N3—C10	1.490 (5)	C19—H19C	0.98
N3—H31N	0.92	S2—O6	1.440 (3)
N3—H32N	0.92	S2—O5	1.465 (3)
C1—C2	1.506 (6)	S2—O4	1.469 (3)
C1—H1A	0.99	S2—C20	1.754 (5)
C1—H1B	0.99	C20—C21	1.372 (6)
C2—H2A	0.99	C20—C25	1.397 (6)
C2—H2B	0.99	C21—C22	1.394 (7)
C3—C4	1.496 (5)	C21—H21	0.95
C3—H3A	0.99	C22—C23	1.406 (7)
C3—H3B	0.99	C22—H22	0.95
C4—C5	1.372 (6)	C23—C24	1.363 (7)
C4—C9	1.379 (6)	C23—C26	1.521 (7)
C5—C6	1.374 (5)	C24—C25	1.390 (6)
C5—H5	0.95	C24—H24	0.95
C6—C7	1.386 (6)	C25—H25	0.95
C6—H6	0.95	C26—H26A	0.98
C7—C8	1.380 (6)	C26—H26B	0.98
C7—C10^i^	1.511 (5)	C26—H26C	0.98
C8—C9	1.391 (5)	S3—O8	1.440 (4)
C8—H8	0.95	S3—O7	1.455 (3)
C9—H9	0.95	S3—O9	1.457 (4)
C10—C7^i^	1.511 (5)	S3—C27	1.766 (5)
C10—H10A	0.99	C27—C32	1.375 (7)
C10—H10B	0.99	C27—C28	1.386 (6)
C11—C12	1.506 (6)	C28—C29	1.383 (7)
C11—H11A	0.99	C28—H28	0.95
C11—H11B	0.99	C29—C30	1.380 (8)
C12—H12A	0.99	C29—H29	0.95
C12—H12B	0.99	C30—C31	1.391 (8)
S1—O2	1.457 (3)	C30—C33	1.521 (8)
S1—O3	1.457 (3)	C31—C32	1.373 (7)
S1—O1	1.463 (3)	C31—H31	0.95
S1—C13	1.765 (5)	C32—H32	0.95
C13—C18	1.381 (6)	C33—H33A	0.98
C13—C14	1.386 (6)	C33—H33B	0.98
C14—C15	1.376 (6)	C33—H33C	0.98
C14—H14	0.95		
			
C12—N1—C1	112.2 (3)	C15—C14—C13	120.1 (5)
C12—N1—H11N	109.2	C15—C14—H14	119.9
C1—N1—H11N	109.2	C13—C14—H14	119.9
C12—N1—H12N	109.2	C14—C15—C16	121.1 (5)
C1—N1—H12N	109.2	C14—C15—H15	119.4
H11N—N1—H12N	107.9	C16—C15—H15	119.4
C2—N2—C3	113.9 (3)	C17—C16—C15	117.7 (5)
C2—N2—H21N	108.8	C17—C16—C19	121.6 (5)
C3—N2—H21N	108.8	C15—C16—C19	120.7 (5)
C2—N2—H22N	108.8	C16—C17—C18	121.8 (5)
C3—N2—H22N	108.8	C16—C17—H17	119.1
H21N—N2—H22N	107.7	C18—C17—H17	119.1
C11—N3—C10	114.6 (3)	C17—C18—C13	119.9 (5)
C11—N3—H31N	108.6	C17—C18—H18	120.1
C10—N3—H31N	108.6	C13—C18—H18	120.1
C11—N3—H32N	108.6	C16—C19—H19A	109.5
C10—N3—H32N	108.6	C16—C19—H19B	109.5
H31N—N3—H32N	107.6	H19A—C19—H19B	109.5
N1—C1—C2	109.0 (3)	C16—C19—H19C	109.5
N1—C1—H1A	109.9	H19A—C19—H19C	109.5
C2—C1—H1A	109.9	H19B—C19—H19C	109.5
N1—C1—H1B	109.9	O6—S2—O5	113.90 (19)
C2—C1—H1B	109.9	O6—S2—O4	113.11 (17)
H1A—C1—H1B	108.3	O5—S2—O4	110.35 (19)
N2—C2—C1	109.8 (3)	O6—S2—C20	107.5 (2)
N2—C2—H2A	109.7	O5—S2—C20	105.69 (19)
C1—C2—H2A	109.7	O4—S2—C20	105.7 (2)
N2—C2—H2B	109.7	C21—C20—C25	119.2 (4)
C1—C2—H2B	109.7	C21—C20—S2	120.8 (4)
H2A—C2—H2B	108.2	C25—C20—S2	120.0 (4)
C4—C3—N2	111.6 (3)	C20—C21—C22	120.4 (5)
C4—C3—H3A	109.3	C20—C21—H21	119.8
N2—C3—H3A	109.3	C22—C21—H21	119.8
C4—C3—H3B	109.3	C21—C22—C23	120.5 (5)
N2—C3—H3B	109.3	C21—C22—H22	119.7
H3A—C3—H3B	108.0	C23—C22—H22	119.7
C5—C4—C9	118.8 (4)	C24—C23—C22	118.4 (5)
C5—C4—C3	120.4 (4)	C24—C23—C26	122.0 (5)
C9—C4—C3	120.7 (4)	C22—C23—C26	119.6 (5)
C4—C5—C6	120.6 (4)	C23—C24—C25	121.5 (5)
C4—C5—H5	119.7	C23—C24—H24	119.2
C6—C5—H5	119.7	C25—C24—H24	119.2
C5—C6—C7	120.9 (4)	C24—C25—C20	120.0 (5)
C5—C6—H6	119.6	C24—C25—H25	120.0
C7—C6—H6	119.6	C20—C25—H25	120.0
C8—C7—C6	119.0 (4)	C23—C26—H26A	109.5
C8—C7—C10^i^	120.6 (4)	C23—C26—H26B	109.5
C6—C7—C10^i^	120.3 (4)	H26A—C26—H26B	109.5
C7—C8—C9	119.4 (4)	C23—C26—H26C	109.5
C7—C8—H8	120.3	H26A—C26—H26C	109.5
C9—C8—H8	120.3	H26B—C26—H26C	109.5
C4—C9—C8	121.2 (4)	O8—S3—O7	114.2 (2)
C4—C9—H9	119.4	O8—S3—O9	110.6 (2)
C8—C9—H9	119.4	O7—S3—O9	111.7 (2)
N3—C10—C7^i^	111.1 (4)	O8—S3—C27	107.7 (2)
N3—C10—H10A	109.4	O7—S3—C27	105.5 (2)
C7^i^—C10—H10A	109.4	O9—S3—C27	106.6 (2)
N3—C10—H10B	109.4	C32—C27—C28	120.0 (5)
C7^i^—C10—H10B	109.4	C32—C27—S3	118.6 (4)
H10A—C10—H10B	108.0	C28—C27—S3	121.3 (4)
N3—C11—C12	109.2 (3)	C29—C28—C27	118.7 (5)
N3—C11—H11A	109.8	C29—C28—H28	120.7
C12—C11—H11A	109.8	C27—C28—H28	120.7
N3—C11—H11B	109.8	C30—C29—C28	122.4 (5)
C12—C11—H11B	109.8	C30—C29—H29	118.8
H11A—C11—H11B	108.3	C28—C29—H29	118.8
N1—C12—C11	110.5 (4)	C29—C30—C31	117.3 (5)
N1—C12—H12A	109.6	C29—C30—C33	121.5 (6)
C11—C12—H12A	109.6	C31—C30—C33	121.2 (6)
N1—C12—H12B	109.6	C32—C31—C30	121.4 (6)
C11—C12—H12B	109.6	C32—C31—H31	119.3
H12A—C12—H12B	108.1	C30—C31—H31	119.3
O2—S1—O3	112.8 (2)	C31—C32—C27	120.1 (5)
O2—S1—O1	110.85 (18)	C31—C32—H32	119.9
O3—S1—O1	111.88 (19)	C27—C32—H32	119.9
O2—S1—C13	107.44 (19)	C30—C33—H33A	109.5
O3—S1—C13	106.6 (2)	C30—C33—H33B	109.5
O1—S1—C13	106.9 (2)	H33A—C33—H33B	109.5
C18—C13—C14	119.4 (5)	C30—C33—H33C	109.5
C18—C13—S1	121.5 (4)	H33A—C33—H33C	109.5
C14—C13—S1	119.1 (4)	H33B—C33—H33C	109.5
			
C12—N1—C1—C2	−169.6 (4)	C14—C13—C18—C17	−1.5 (7)
C3—N2—C2—C1	−172.3 (4)	S1—C13—C18—C17	−178.9 (3)
N1—C1—C2—N2	−172.0 (4)	O6—S2—C20—C21	7.7 (4)
C2—N2—C3—C4	−51.8 (5)	O5—S2—C20—C21	129.6 (4)
N2—C3—C4—C5	−57.0 (6)	O4—S2—C20—C21	−113.4 (4)
N2—C3—C4—C9	119.2 (4)	O6—S2—C20—C25	−172.8 (4)
C9—C4—C5—C6	−1.2 (7)	O5—S2—C20—C25	−50.8 (4)
C3—C4—C5—C6	175.1 (4)	O4—S2—C20—C25	66.2 (4)
C4—C5—C6—C7	1.1 (7)	C25—C20—C21—C22	0.7 (8)
C5—C6—C7—C8	0.7 (7)	S2—C20—C21—C22	−179.7 (4)
C5—C6—C7—C10^i^	−178.6 (4)	C20—C21—C22—C23	−1.0 (8)
C6—C7—C8—C9	−2.4 (7)	C21—C22—C23—C24	0.5 (8)
C10^i^—C7—C8—C9	176.9 (4)	C21—C22—C23—C26	179.9 (5)
C5—C4—C9—C8	−0.5 (7)	C22—C23—C24—C25	0.2 (8)
C3—C4—C9—C8	−176.8 (4)	C26—C23—C24—C25	−179.2 (5)
C7—C8—C9—C4	2.3 (7)	C23—C24—C25—C20	−0.4 (8)
C11—N3—C10—C7^i^	51.9 (5)	C21—C20—C25—C24	−0.1 (7)
C10—N3—C11—C12	177.8 (4)	S2—C20—C25—C24	−179.6 (4)
C1—N1—C12—C11	165.6 (4)	O8—S3—C27—C32	84.8 (5)
N3—C11—C12—N1	170.9 (4)	O7—S3—C27—C32	−152.9 (4)
O2—S1—C13—C18	−119.5 (4)	O9—S3—C27—C32	−33.9 (5)
O3—S1—C13—C18	119.3 (4)	O8—S3—C27—C28	−95.0 (4)
O1—S1—C13—C18	−0.5 (4)	O7—S3—C27—C28	27.4 (5)
O2—S1—C13—C14	63.1 (4)	O9—S3—C27—C28	146.3 (4)
O3—S1—C13—C14	−58.1 (4)	C32—C27—C28—C29	1.0 (7)
O1—S1—C13—C14	−177.8 (3)	S3—C27—C28—C29	−179.3 (4)
C18—C13—C14—C15	1.0 (7)	C27—C28—C29—C30	−0.8 (8)
S1—C13—C14—C15	178.4 (3)	C28—C29—C30—C31	1.2 (9)
C13—C14—C15—C16	0.1 (7)	C28—C29—C30—C33	178.5 (6)
C14—C15—C16—C17	−0.6 (7)	C29—C30—C31—C32	−1.7 (10)
C14—C15—C16—C19	−179.4 (4)	C33—C30—C31—C32	−179.1 (6)
C15—C16—C17—C18	0.1 (7)	C30—C31—C32—C27	2.0 (9)
C19—C16—C17—C18	178.9 (4)	C28—C27—C32—C31	−1.5 (8)
C16—C17—C18—C13	1.0 (7)	S3—C27—C32—C31	178.7 (5)

Symmetry codes: (i) −*x*+1, −*y*+1, −*z*+1.

### Hydrogen-bond geometry (Å, °)

**Table:**

*D*—H···*A*	*D*—H	H···*A*	*D*···*A*	*D*—H···*A*
N1—H11N···O7^i^	0.92	1.86	2.738 (5)	159
N1—H12N···O8	0.92	2.10	2.923 (6)	148
N1—H12N···O9	0.92	2.33	3.130 (5)	145
N2—H21N···O5	0.92	1.85	2.745 (5)	164
N2—H22N···O1^ii^	0.92	1.83	2.706 (4)	159
N3—H31N···O4^ii^	0.92	1.84	2.748 (4)	168
N3—H32N···O2	0.92	1.92	2.842 (5)	177
N3—H32N···O1	0.92	2.58	3.081 (4)	115

Symmetry codes: (i) −*x*+1, −*y*+1, −*z*+1; (ii) −*x*+2, −*y*+1, −*z*+1.
